# An Original Number

**Published:** 1859-08

**Authors:** 


### EDITORIAL AND MISCELLANEOUS.

### AN ORIGINAL NUMBER.

We hope our friends will not be deterred from sending on their contributions for our journal, because we had no space in the July number for selected matter. We desire to keep a full drawer, and this can only be done by regular and constant supplies.

It we could only have the privilege of spreading before the profession through our paper, one hundredth part of the talent, learning and practical skill of the medical men of the south, we should want no better resources, from which to draw material to sustain a successful medical journal.

